# Comprehensive mass spectrometric metabolomic profiling of a chemically diverse collection of plants of the Celastraceae family

**DOI:** 10.1038/s41597-024-03094-6

**Published:** 2024-04-22

**Authors:** Luis-Manuel Quiros-Guerrero, Pierre-Marie Allard, Louis-Felix Nothias, Bruno David, Antonio Grondin, Jean-Luc Wolfender

**Affiliations:** 1 Institute of Pharmaceutical Sciences of Western Switzerland, University of Geneva, CMU, 1211 Geneva, Switzerland; 2 School of Pharmaceutical Sciences, University of Geneva, CMU, 1211 Geneva, Switzerland; 3 Department of Biology, University of Fribourg, 1700 Fribourg, Switzerland; 4 Green Mission Department, Herbal Products Laboratory, Pierre Fabre Research Institute, Toulouse, France

**Keywords:** Mass spectrometry, Cheminformatics, Natural products, Metabolomics, Cheminformatics

## Abstract

Natural products exhibit interesting structural features and significant biological activities. The discovery of new bioactive molecules is a complex process that requires high-quality metabolite profiling data to properly target the isolation of compounds of interest and enable their complete structural characterization. The same metabolite profiling data can also be used to better understand chemotaxonomic links between species. This Data Descriptor details a dataset resulting from the untargeted liquid chromatography-mass spectrometry metabolite profiling of 76 natural extracts of the Celastraceae family. The spectral annotation results and related chemical and taxonomic metadata are shared, along with proposed examples of data reuse. This data can be further studied by researchers exploring the chemical diversity of natural products. This can serve as a reference sample set for deep metabolome investigation of this chemically rich plant family.

## Background & Summary

The Celastraceae family (Q135336), also known as the ‘*bittersweet*’ family, englobes approximately 98 genera and 1350 species of shrubs, vines, and trees^1^. It has a nearly worldwide distribution, from tropical to temperate regions, except in the Arctic^2^. Many species have been used in traditional medicine due to their broad range of bioactivities^3,4^, like immunosuppression^5^, antiprotozoal^6^, antiproliferative^7^, anticancer^8,9^, and antimicrobial^10^, which are linked to the chemical diversity of natural products (NP) present in these plants^3,8,11–13^. The three best-known compounds correspond to maytansine (Q6720157), a potent maytansinoid that targets microtubules and induces mitotic arrest^14–16^; triptolide (Q906351), a diterpenoid tri-epoxide with proven effects against lupus, cancer and rheumatoid artritis^3,17–20^; and celastrol (Q5057534), a nor-triterpene quinone methide studied for its potential to treat inflammatory and autoimmune diseases. Triptolide and celastrol were isolated from the Thunder god vine (*Tripterygium wilfordii* Hook F., Q1424919)^21–23^. The chemical variety and extensive array of biological activities exhibited by this family underscore the value of conducting thorough metabolome investigations of representative species.

Today, plant metabolomes can be efficiently characterized through untargeted Ultra-High-Performance Liquid Chromatography-High-Resolution data-dependent tandem Mass Spectrometry (UHPLC-HRMS^2^). This provides HRMS and corresponding MS^2^ spectra on most detected ions^24–27^. The aligned MS feature tables from UHPLC-HRMS^2^ facilitate the detection of variations in the metabolite composition between samples, while annotation/dereplication strategies provide an *in-depth* overview of the chemistry linked to each organism^28,29^.

In the context of UHPLC-HRMS^2^, the clear identification of NPs within mixtures faces challenges due to the limited scope of metabolomics Data Bases (DB, *e.g*. lack of experimental spectra). This results in difficulty matching known compounds, while structural similar matches lead to uncertain or partial identifications^30–32^. Moreover, within a lab, the absence of standardized reference compounds can further complicate the validation of putative annotations.

To enhance the accuracy of NP dereplication^33^, the spectral similarity networking approach allows the organization of spectral MS^2^ data through molecular networking (MN). In the MN, each node corresponds to a feature (specific mass-to-charge ratio (*m/z*) value observed at a given retention time (RT)), and the edges between nodes are drawn based on the similarity of their MS^2^ profiles. The MN approach is useful to analyze and visualize relationships between features in the samples^26,34^, and can be easily performed on the Global Natural Product Social Molecular Networking platform (GNPS)^26^. This approach also involves the comparison of experimental spectra provided by the scientific community within the GNPS platform. This comparison of experimental MS^2^ with those in the DB is facilitated by spectral scoring algorithms, generating a matching score that reflects the similarity between a query MS^2^ spectrum and a metabolite’s MS^2^ spectrum within the DB^31,35^. However, caution is required as this information is highly instrument-dependent (*e*.*g*. LC conditions, ionization type, MS acquisition mode, collision energy, among others)^31^.

Given that only approximately 10% of features are commonly annotated using experimental spectral DB alone (*e.g*. GNPS), several computational tools have emerged to bridge this gap by enabling metabolite annotation through *in silico* spectrum prediction and structural candidates identification^32,36,37^. These tools serve as a link between MS^2^ spectra and molecular structure DB. Broadly, two distinct approaches are employed: (1) predicting an *in silico* MS^2^ spectrum based on a molecular structure, and (2) predicting a spectral fingerprint from an MS^2^ spectrum and then matching it to a molecular structure from DBs^31^. The first approach is the base for the strategy developed by Allard *et al*.^24^, allowing the integration of NPs *in silic*o DB (ISDB) and MN^26^ in the dereplication pipeline. An additional improvement in this strategy was introduced by Rutz *et al*.^25^ by considering the taxonomic distance between the candidate structure’s biological source and the annotated Natural extracts’s (NE) biological sources. This type of approach was automated (*e.g*., TimaR). This was shown to systematically improve the annotation results quality of several computational metabolite annotation approaches (ISDB^24^, Sirius^38^, among others). The comparison of experimental and *in silico* MS^2^ spectra is done through spectral similarity algorithms. This approach is the one used in the GNPS platform. The second approach, *fingerprint matching*, is the basis of the Sirius workflow^38^ and its dependencies. It starts by predicting the molecular formula (MF) of a feature using precursor *m/z*, isotope pattern, and MS^2^ spectrum. These MF candidates are further refined by the ZODIAC module^39^ and then used to find potential structures from DB. Then, the CSI:FingerID algorithm generates molecular fingerprints from the experimental MS^2^ spectra by using support vector machine models^40^. These molecular fingerprints are used to identify potential molecular structures by matching them in DBs like KEGG^41^, HMDB^42^, and PubChem^43^. Because MS annotations rely on MF and fragmentation patterns, this form of spectrometric data primarily emphasizes the atoms’ connectivity. However, it does not provide information about spatial configuration. As a result, any structural annotations generated using these annotation methods are presented as putative 2D planar structures. Determining the 3D structure often involves making hypotheses or inferences, particularly when dereplication outcomes identify a constituent that has already been described and fully characterized within the same botanical species or taxonomically related samples^44^. The CANOPUS module from Sirius systematically annotates the chemical classes directly from the MS^2^ spectral fingerprint without the necessity of a formal structural annotation^45^. The chemical class taxonomy is based on NPClassifier, a deep neural network-based NP classification tool^46^. This tool produces a classification structured into three hierarchical levels (pathway, superclass, and class), which are determined based on expert knowledge^46^.

Additionally, during UHPLC-HRMS^2^ ionization, a metabolite can generate multiple ion species (*e.g*. [M + H]^+^, [M + Na]^+^ or [M + K]^+^ adducts). Multiple HRMS-MS^2^ feature pairs are obtained for a metabolite. This can lead to unwanted separation of molecular families (subnetworks) and limits library annotations across MN. The development of Ion Identity MN (IIMN) addressed this by merging features of identical molecules into groups (Ion Identity Networks, IIN) based on MS^1^ feature peak shape correlation. This simplifies comparisons and enhances metabolite composition representation^47^. The integration of machine learning-driven *in-silico* annotation tools has proven highly effective for characterizing metabolites across NPs exploration^32,35^. Employing a combination of diverse annotation approaches and strategies like IIMN offers better coverage across the chemical space of the samples leading to a more comprehensive description.

This Data Descriptor provides information on a UHPLC-HRMS^2^ dataset of 76 NEs from the Celastraceae family^48^, and the dereplications results obtained through different annotation strategies (GNPS^26^, ISDB^24,25^, Sirius^38^, and CANOPUS^45^). The set is part of the Pierre Fabre Laboratories (PFL) plant collection. The PLF from 1998 to 2015 conducted high-throughput screening (HTS)-research on the discovery of bioactive NP as plant anticancer agents. Their NEs HTS program (stopped in 2015), ended up with *c.a*. 17,000 unique specimens of plant parts, which made it one of the largest private collections in the world^49^.

The European Commission assigned the accession number 03-FR-2020 to the PFL collection on April 2020^50^, certifying that the collection meets the EU Access and Benefit-Sharing (ABS) Regulation criteria, and fulfils the requirements of the Nagoya Protocol^51^. To date, only 3 European collections are recognized^50^. The EU ABS Regulation, Regulation (EU) No 511/2014, establishes a comprehensive framework for the fair and equitable utilization of genetic resources and associated traditional knowledge within the European Union. The regulation mandates the obtention of prior informed consent from provider countries before accessing genetic resources and requires the establishment of mutually agreed terms between users and providers^50,51^.

This Celastraceae set is composed of 36 species of 14 different genera and several plant parts that were analyzed for each species, totalizing 76 samples. The size of the set was restricted to the samples available in the PFL collection. It contains about 15% of all genera in the family. In the Celastraceae family, as documented by the LOTUS initiative^52^, which consolidates a significant portion of prior phytochemical public knowledge, data is available for 164 species (*c*.*a*. 12% of all species) from 36 genera (*c*.*a*. 37%), with each genus having at least one reported compound. This set covers approximately 30% of these studied genera, and additionally, three genera have not been phytochemically studied to our knowledge: *Mystroxylon* (Q9047958, WD query), *Evonymopsis* (Q151181. WD query) and *Loeseneriella* (Q9023498, WD query). The annotation results obtained from this set, as detailed in the Technical Validation section, offer a rather broad overview of the known Celastraceae family chemistry. The coverage of chemical classes, particularly agarofurans sesquiterpenoids, friedelane, and lupane triterpenoids, suggests that the dataset matches most of the reported compounds.

The set underwent analysis on an analytical platform capable of conducting UHPLC-HRMS^2^ metabolite profiling in both positive (PI) and negative (NI) ionization modes. Complementary data were also recorded using a Photo Diode Array (PDA) and a Charged Aerosol Detector (CAD). The CAD provides numerous benefits, including universal detection, sensitivity, robustness, versatility, and compatibility^53,54^. The incorporation of semiquantitative detectors in NP chemistry offers a practical and cost-effective method for screening, analyzing, and characterizing NEs^55,56^. Depending on the scope of the study, the availability of semiquantitative data assists in rapidly identifying features of interest and assessing whether the isolation of such compounds is worthwhile. Furthermore, semiquantitative detectors allow for a more detailed examination of the actual composition of a sample, providing a closer and more comprehensive understanding of its chemical constitution.

The data (UHPLC-PDA-CAD-HRMS^2^ analyses, and dereplication results) for the Celastraceae set is publicly available on the MassIVE data repository with the accession number MSV000087970. It is linked with the GNPS platform^26^ through this interactive dashboard^57^. The metadata is compatible with the GNPS/ReDU requirements allowing easy re-utilization. It includes the general details of each sample like taxonomy, type of sample, and plant part^26,58^. It contains the most recent ‘accepted’ names for each organism obtained from the Open Tree of Life (OTL v13.4)^59^.

**Fig. 1:**
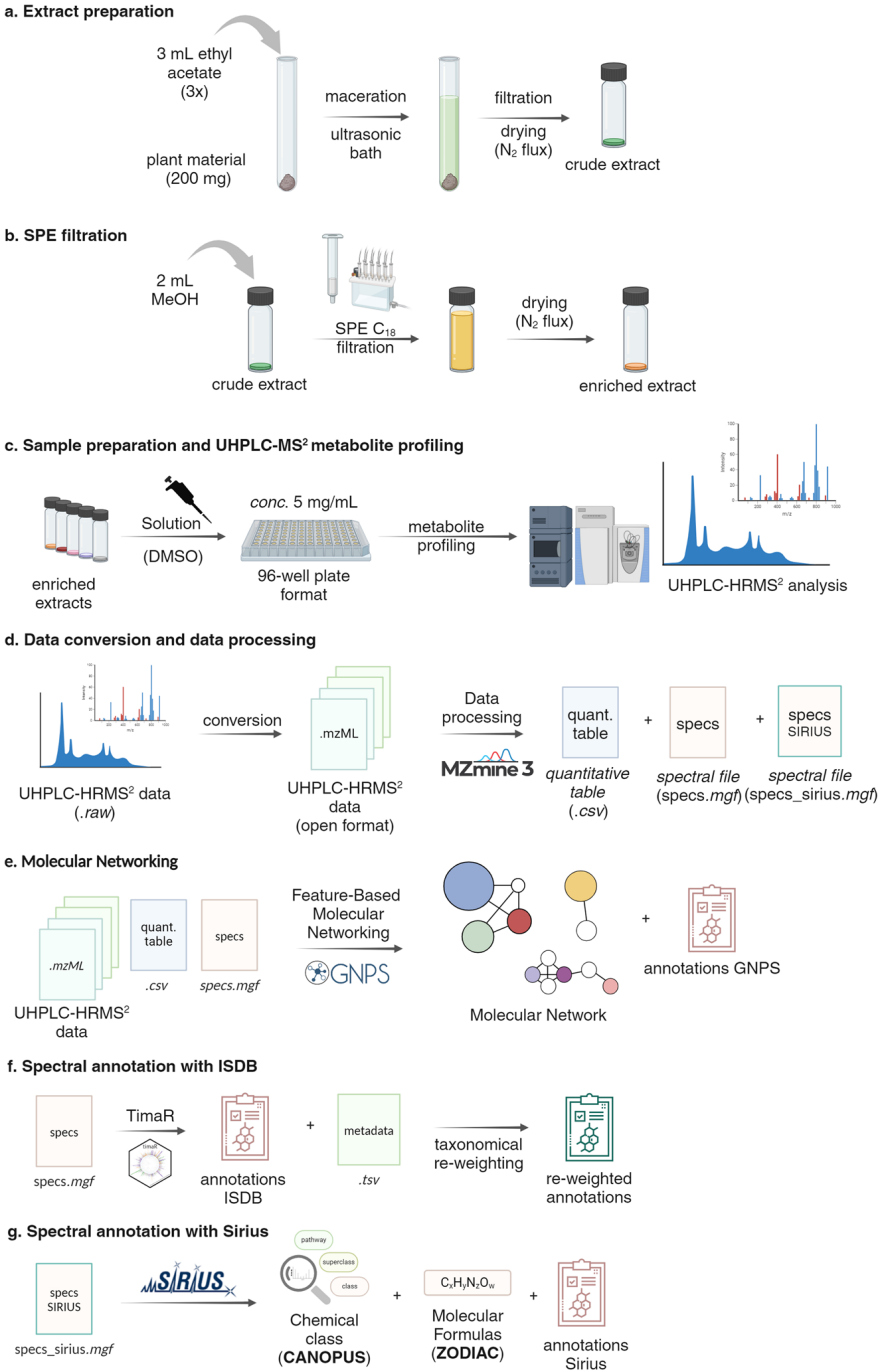
Workflow used for the UHPLC-PDA-CAD-ESI-HRMS^2^ metabolite profiling and metabolite annotation of the Celastraceae set. (**a**) **Extract preparation**: To obtain the crude extract, each sample underwent an ethyl acetate maceration assisted by ultrasound bath, filtration and drying under N_2 _flux three-step extraction process. (**b**) **SPE filtration**: To remove highly lipophilic compounds, the crude extracts were dissolved in methanol and filtered through a C_18_ SPE cartridge. The filtrate was dried under N_2_ flux to obtain an enriched extract. (**c**) **Sample preparation and UHPLC-MS**^**2**^ **metabolite profiling**: The enriched extracts were dissolved in DMSO to a concentration of 5 mg/mL and a 100 *μ*L aliquot was transferred (without further dilution) to a 700 *μ*L 96-well plate for UHPLC-PDA-CAD-HRMS^2^ metabolite profiling. (**d**) **Data conversion and data processing**: The.*raw* data files were converted to the*.mzML* open-source format. The converted data was analyzed using MZmine3 to generate a ‘quantitative table.*csv*’ of features (peak with *m/z*@RT) present in the data set, as well as a spectral file (specs.*mgf*) that includes the MS^2^ spectral information for each feature. (**e**) **Molecular Networking**: Upon conversion, the*.mzML* files, along with the quantitative table.*csv* and spectral specs.*mgf* file, were uploaded to the GNPS website. An FBMN was generated and an automated search for spectral matches against experimental DBs was performed. (**f**) **Spectral annotation with ISDB**: The spectral specs.*mgf* file was subjected to a taxonomically informed metabolite annotation pipeline (ISDB annotation followed by taxonomical reweighting) using TimaR. (**g**) **Spectral annotation with Sirius**: The specs_sirius.*mgf* file was processed with Sirius to obtain the chemical classes, molecular formulas, and structural annotations with CSI-FinguerID, and CANOPUS.

The general procedures used to generate the data set, from the production of the NE extract to the MS^2^ spectral annotations results are detailed in Fig. 1. Selective extraction using ethyl acetate was employed targeting compounds of intermediate polarity (which are those in general having drug-likeness properties) and subsequently, filtration through a C_18_ SPE cartridge was conducted to eliminate the highly lipophilic compounds (Fig. 1a,b).

The resulting extracts were dissolved in DMSO and subjected to UHPLC-PDA-CAD-ESI-HRMS^2^ metabolite profiling. DMSO effectively dissolves compounds with various polarities, maintains solution stability, prevents evaporation, and it is compatible with biological assays, making it an ideal solvent for NEs dissolution, even at higher concentrations. Additionally, during UHPLC metabolite profiling, DMSO exhibited no specific issues, suggesting its suitability for analytical purposes given the small injection volume (1–2 *μ*L). This enables using the same solution for metabolite profiling and biological screening, enhancing correlation between HRMS^2^ data and screening results. The extract solutions in DMSO were kept at −20 °C after analysis for preservation.

The acquired data was transformed into.*mzML* open format^60^, followed by processing using MZmine3^61,62^ (Fig. 1c,d). The processed data then underwent a dereplication workflow, which involved organizing spectra through Feature-based MN (FBMN)^34^ (Fig. 1e) and *in silico* annotations (Fig. 1f,g). These three steps are executed in parallel. To construct the FBMN and perform the ISDB^24^ *in silico* annotations, the ‘specs.*mgf*’ file and ‘quantitative features table.*csv*’ were exported through the ‘Molecular networking files’ module in MZmine3. Additionally, for Sirius^38^ *in silico* annotations, a second ‘specs_sirius.*mgf*’ file was exported using MZmine3’s module ‘SIRIUS/CSI-FingerID’ (Fig. 1g).

After the generation of the FBMN, the spectral annotation was done in the first instance with the automatic identification of all recorded MS^2^ spectra against the public DB of GNPS. This was followed by two independent *in silico* annotation strategies mentioned above. The set was subjected to the taxonomically informed metabolite annotation pipeline (ISDB^24^ annotation followed by taxonomical reweighting^25^), using TimaR. The NPs *in silico* spectral DB used was based on the structures gathered in the LOTUS initiative (v4.0)^52^ and the Dictionary of Natural Products (DNP). The set was also submitted to Sirius^38^ to obtain accurate MFs, and structural annotations through CSI:FingerID^40^, followed the CANOPUS^45^ chemical class assignment. To ensure a minimum quality of all the putative identifications, the annotations were filtered and consolidated using one of the modules of Inventa^63^ script according to each annotation pipeline. The GNPS annotations were filtered based on the ppm error, the number of shared peaks, the cosine score, and the ionization mode. The Sirius annotations were filtered based on the Zodiac and Confidence scores. Similarly, the chemical classes from CANOPUS were considered only if the Class confidence was higher than the established threshold (see *Methods*). According to the guidelines proposed by the Metabolomics Standards Initiative (MSI)^64, 65^, the annotation levels of the putative identities assigned to the data include MSI Level 2a for library-based annotations using GNPS^26^, and MSI Level 3 for *in silico* annotation tools such as CSI:FingerID^40^, CANOPUS^45^, and ISDB^24^.

To explore the spectral diversity of the data set, a visualization of the FBMN was constructed using Cytoscape^66^. Different layers of information were added according to the chemical taxonomy (color according to the chemical class, superclass, and pathway), and to the proportion of ions in each sample (ratio of MS intensity for each feature detected in the different samples). The raw and filtered annotations, and the Cytoscape files, for PI and NI, can be found in this MassiVE repository link.

## Methods

### Plant material

The plant material of all the samples was provided as dry-grounded powder by the PFL laboratories. The individual PFL identifiers (code V1XXXXX) for each sample can be found in the metadata information in the MassIVE repository. Details regarding the collection, drying, and preservation of samples for the overall PFL collection were described by Allard *et al*.^49^.

### Maceration and sample preparation

A mass of 200 mg of dry plant material was extracted three times with 3 mL of ethyl acetate (Fisher Chemicals, Reinach, Switzerland) in an ultrasound bath (30 minutes each time). The total solvent volume (*c.a*. 9 mL) was filtered through a paper filter and dried under an N_2_ flux. The residue was dissolved in methanol (2 mL) and passed through a pre-conditioned (according to the manufacturer) C_18_ SPE cartridge (1000 mg). The filtrate and the following washing (1 mL of 1:1 MeOH:EtOAc) were collected in the same vial. The solvent was dried under an N_2_ flux. The enriched extract was solubilized in DMSO (Sigma, St Louis, USA) at a concentration of 5 mg/mL. See Fig. 1a–c.

### UHPLC-HRMS^2^ metabolite profiling

Samples were analyzed using an Acquity I-Class UPLC-PDA system (Waters Co., Milford, MA, USA) coupled to a Q-Exactive Focus mass spectrometer (Thermo Scientific, Bremen, Germany) equipped with a heated electrospray ionization source (HESI-II). The chromatographic separation was done on a Waters BEH C_18_ column (50 × 2.1 mm i.d., 1.7 *µ*m, Waters Co., Milford, MA, USA), using a linear gradient from 5 to 100% B over 7 min, at a 600 *µ*L/min flow rate. The solvent system consisted of [A]: water with 0.1% formic acid and [B]: acetonitrile with 0.1% formic acid. The injection volume was 2 *µ*L, and the column was kept at 40 °C. The mass spectrometry parameters were as follows for PI mode (NI mode): spray voltage at + 3.5 kV (−2.5 kV); heater temperature at 220 °C; the capillary temperature at 350.00 °C; S-lens RF at 45 (arb. units); sheath gas flow rate at 55 (arb. units) and auxiliary gas flow rate at 15.00 (arb. units). The system was also coupled to a Charged Aerosol Detector (Thermo Scientific^TM^, Bremen, Germany) kept at 40 °C. The PDA detector wavelength range was set from 210 nm to 400 nm with a resolution of 1.2 nm. The instruments were controlled using Thermo Scientific Xcalibur 3.1 software. The mass acquisition events were programmed as follows: one full scan with a resolution of 35,000 FWHM (at *m/z* 200) followed by three (top 3) centroid data-dependent MS^2^ (dd-MS^2^) at a resolution of 17,500 FWHM (100 to 1500 *m/z* range). Each dd-MS^2^ scan acquisition event was done in discovery mode using the Apex trigger mode (2 to 7 s), a dynamic exclusion of 2.0 s with an isolation window of 1.5 Da, and a stepped normalized collision energy (NCE) of 15, 30, and 45 units. Additional parameters were set as follows: default mass charge: 1; Automatic gain control (AGC) target 2.0E^5^; Maximum IT: 119 ms; Loop count: 3; Min AGC target: 2.6E^4^; Intensity threshold: 1.

The injection order for the 76 sets of extracts was randomized. Each set was measured in batches of ten samples, with each batch separated by one blank (solvent) injection, one QC sample, and a second blank injection. The so-called QC sample corresponded to a pooled mix of all 76 extracts. See Fig. 1c.

### Data processing with MZmine3

The raw data were converted to.*mzXML* open format with the MS Convert software, part of the ProteoWizard^60^ project. The converted files were uploaded and processed with MZmine3^61,62^. The parameters for processing were as follows in PI (NI) mode: MS^1^ level mass detection 1.0E^6^ (1.0E^5^). MS^2^ detection noise level was set to 0.00 for both ionization modes. The chromatograms were built using the ADAP chromatogram algorithm (minimum group size in *number* of scans, 4; group intensity threshold, 1.0E^6^ (1.0E^5^ negative); minimum highest intensity, 1.0E^6^ (1.0E^5^ negative), scan-to-scan accuracy (*m/z*) of 0.0020 or 10.0 ppm). Deconvolution was made with the ADAP feature resolver algorithm (S/N threshold, 30; minimum feature height, 1.0E^6^ (1.0E^5^); coefficient area threshold, 110; peak duration range, 0.01–1.0 min; RT wavelet range, 0.01–0.08 min). Isotopes were detected using the 13 C isotope filter (*m/z* tolerance of 0.0050 or 8.0 ppm, RT tolerance of 0.03 min (absolute), the maximum charge set at 2, and the representative isotope used was the lowest *m/z*). The feature lists were filtered by RT (PI: 0.70–8.00 min, NI: 0.40–8.00 min), and only ions with an associated MS^2^ spectrum were kept, before alignment. The join-aligner algorithm was used for alignment (*m/z* tolerance, 0.0050 or 8.0 ppm; RT tolerance, 0.05 min). The aligned feature table was filtered to remove duplicates (*m/z* tolerance, 8.0 ppm; RT tolerance, 0.10 min) and features present in the corresponding blanks. The resulting filtered lists were subjected to Ion Identity Networking^47^ (metaCorrelate module: RT tolerance, 0.10 min; minimum height, 1.0E^5^ (1.0E^3^); Intensity correlation threshold 1.0E^5^ (1.0E^3^) and the Correlation Grouping with the default parameters. Ion identity networking: *m/z* tolerance, 8.0 ppm; check: one feature; minimum height: 1.0E^5^ (1.0E^3^), annotation library [maximum charge, 2; maximum molecules/cluster, 2; Adducts:[M + H]^+^, [M + Na]^+^, [M + K]^+^, [M + NH_4_]^+^, [M + 2H]^2+^ ([M-e]^−^, [M-H]^−^, [M-2H + Na]^−^, [M + Cl]^−^, [M + FA]^−^). Modifications (PI and NI): [M-H_2_O], [M-2H_2_O], [M-CO_2_], [M + HFA], [M + ACN]. Annotation refinement: Delete small networks without major ion, yes; Delete networks without monomer, yes; Add ion identities networks: *m/z* tolerance: 8 ppm; minimum height: 1.0E^5^ (1.0E^3^). Annotation refinement: Minimum size: 1; Delete small networks without major ion: yes; Delete small networks: Link threshold, 4; Delete networks without monomer: yes. Check all ion identities by MS^2^: *m/z* tolerance (MS^2^): 10 ppm; min-height in MS^2^: 1.0E^3^ (1.0E^3^); Check for multimers: yes; Check neutral losses (MS^1^- > MS^2^): yes. The resulting aligned peak lists were exported using the GNPS-Feature Based Molecular Networking and Sirius/CSI FIngerID modules. The batch files for both modes can be found here (See Fig. 1d).

### Spectral organization through Feature-Based Molecular networking

The Feature-Based Molecular Networking analyses, for both ionization modes, were created with the default parameters on the GNPS website (documentation). The precursor ion mass tolerance was 0.02 Da with an MS^2^ fragment ion tolerance of 0.02 Da. The edges were filtered for a cosine score above 0.7 and at least 6 matched peaks. For the GNPS automatic library search, all matches were required to have a score above 0.6, and at least three matched peaks. The jobs can be found here: PI and NI (See Fig. 1e).

### ISDB annotation and taxonomically informed reponderation

The taxonomically informed metabolite annotations were made using TimaR^24,67^, following the documentation in this repository (v 2.4.0) and re-ranking from the taxonomical information available on LOTUS^52^. The ISDB used for this process includes the combined records of the Dictionary of Natural Products (DNP, v 30.2) and the LOTUS Initiative records (v4.0) (See Fig. 1f).

### Annotation using Sirius

The sirius_specs.*mgf* file exported from MZmine was processed with Sirius^38^ (v 5.5.5) command-line tool on a Linux server. The parameters used were *Possible ionizations*: [M + H]^+^, [M + NH_4_]^+^, [M-H_2_O + H]^+^, [M + K]^+^, [M + Na]^+^, ([M-H]^−^, [M + Cl]^−^, [M + Br]^−^); *Instrument profile*: Orbitrap; *mass accuracy*: 5 ppm for MS^1^ and 7 ppm for MS^2^, the DB for molecular formulas and structures: BIO and custom DBs (LOTUS, Dictionary of Natural Products), *maximum m/z to compute*: 1000. To improve the prediction of the molecular formulas, the ZODIAC score threshold was set to 0.99^39^. CSI: FingerID^40^ was used for structure prediction (the significance was computed with COSMIC^68^). The prediction of the chemical class was made with CANOPUS^45^ using the NPClassifier taxonomy^46^ (See Fig. 1g). The custom DBs were generated as described in the documentation directly from the frozen metadata of the LOTUS Initiative^52^ and the DNP (v 30.2) private file from our laboratory.

### General annotations quality filtering

The filtering of the annotation results was done using a script part of the *Inventa*^63^. For the filtering of the GNPS annotations, the following parameters were established: max_ppm_error: 5, shared_peaks: 10, min_cosine: 0.6, ionization_mode: ‘pos’, max_spec_charge: 2. For the filtering of the ISDB annotations, the following parameters were used: min_score_final: 0.3, min_ZODIACScore: 0.9, and min_ConfidenceScore: 0.25. Only chemical classes with a min_class_confidence of 0.8 were further considered.

## Data Records

The raw and.*mzXML* UHPLC-HMRMS^2^ data^48^ of all the samples (NEs and QCs) are accessible via MassIVE with the accession number MSV000087970. This repository is organized into four main folders: RAW, mzML, Metadata, and Other. The ‘RAW’ and ‘mzML’ folders include subfolders labeled 01, 02, and 03, each representing analytical replicates. Within these, there are two subfolders, one for each ionization mode, (*e.g*. 01_POS and 01_NEG). The ‘Metadata’ folder contains information related to each sample, including its type and taxonomy, as well as the ReDU metadata, necessary for reusing public data in the GNPS environment. The ‘Other’ folder houses various annotation results from Sirius, CANOPUS, and ISDB in both PI and NI modes. Additionally, this folder contains the.*cys* Cytoscape file, and the MZmine batch parameters files for both ionization modes.

The molecular network for PI and NI modes can be accessed following the GNPS hyperlinks: (PI) https://gnps.ucsd.edu/ProteoSAFe/status.jsp?task=8156bf469f1e4b8a8fe602b9b1d5c635 and (NI) https://gnps.ucsd.edu/ProteoSAFe/status.jsp?task=d477f360ddb344a593b935624782d8eb. The ISDB and Sirius annotations (CSI:FingerID and CANOPUS) are available for both ionization modes through this link.

## Technical Validation

### Assessment of the quality of the UHPLC-HRMS^2^ measurements

**Fig. 2:**
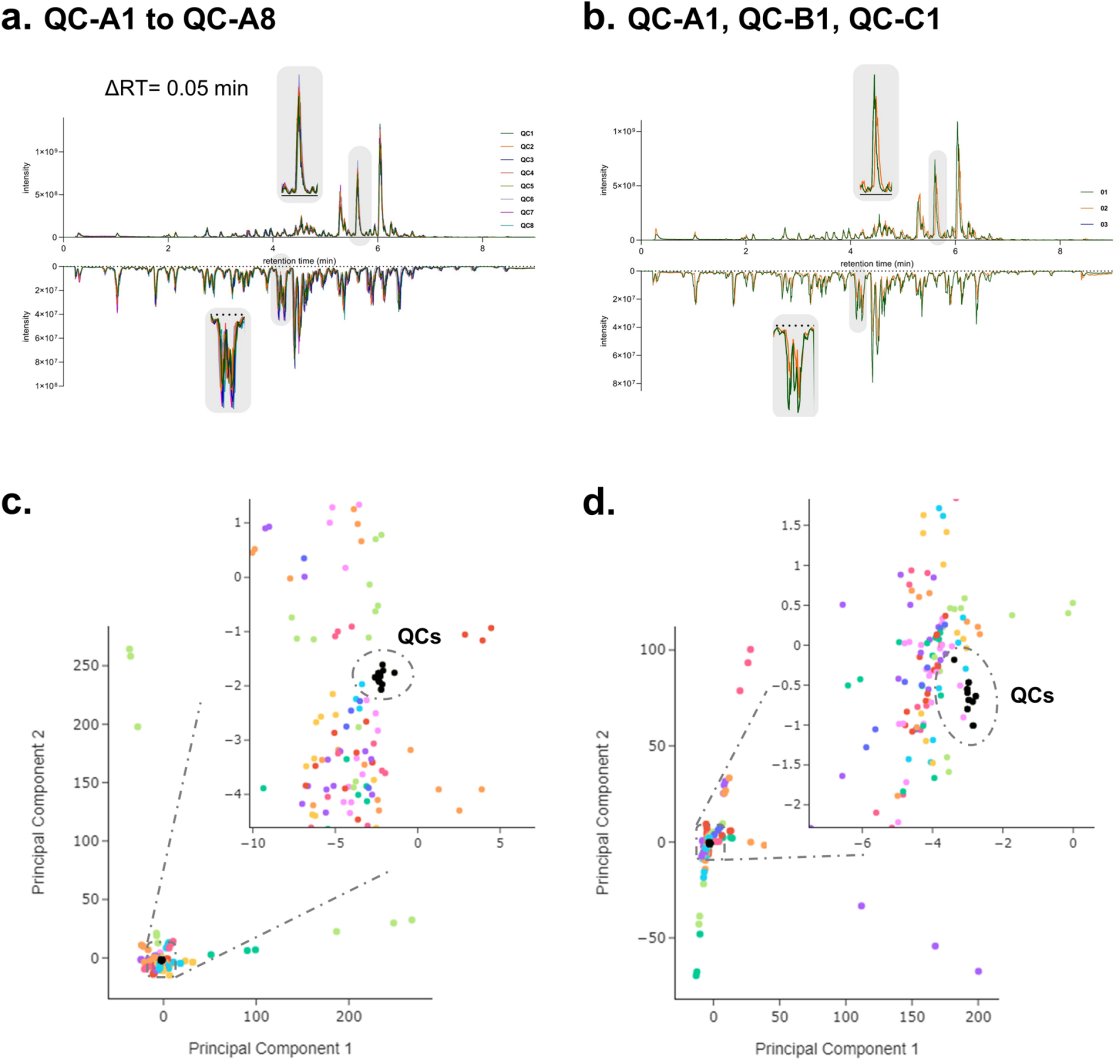
Comparative analysis of UHPLC-HRMS^2^ metabolite profiles in PI and NI for (**a**) the eight QC technical replicates (QC-A1 to QC-A8) in analytical replicate A. (**b**) The three analytical replicates of the QC technical replicate 1 (QC-A1, QC-B1, and QC-C1). Principal Component Analysis (PCA) projections for the combined results of the three analytical replicates including the QCs in PI (**c**) and NI (**d**). The projections were based on the quantitative table generated using MZmine3. The color scheme corresponds to the analytical replicates (injections A-C) in both ionization modes. The enlarged regions show the clustering of the QCs in the projection (colored in black).

The experimental design included eight individual quality controls, designated as QC1 through QC8, which were composed of pooled samples (a combination of all 76 NEs). The objective was to assess the uniformity and constancy of the UHPLC-HRMS^2^ metabolite profiling runs. The QCs were injected following batches of ten consecutive samples, resulting in a total of eight QCs being injected once within the set (QC1 to QC8). The entire set, comprising samples, blanks, and QCs, underwent three rounds of measurements denoted as injections A, B, and C. This entailed a cyclical analysis of the complete set, with alternation between PI and NI modes. After data processing in MZmine3, the response areas and retention times of all peaks in the aligned features table of only QC samples (all technical replicates (QC1–8) in the three analytical replicates (injections A, B, and C) were compared. This showed that the detected features exhibited consistent retention times, with a maximum deviation of Δ 0.05 minutes across all injections for both ionization modes (see Fig. 2).

The variation of the total sum area (addition of the areas of all features detected in a sample) between the eight QCs technical replicates (1–8) within each analytical replicate (injection A, B, and C) ranged from 3% to 8% for PI and from 4% to 7% for NI (see Fig. 2a). Comparison of the total sum area of the same QCs technical replicates between analytical replicates (*e.g*. QC-A1, QC-B1, QC-C1) showed a variation range from 2% to 7% for the PI and 6% to 9% for NI (see Fig. 2b). Additionally, a visual assessment of the peak areas from all the QCs across the three analytical replicates of the dataset was conducted using an interactive heat map plot (PI, NI). A Principal Component Analysis (PCA) constructed with the complete dataset in both ionization modes, validated these findings. The PCA plots exhibited defined clustering of all QCs samples and distinct separation of the diverse NEs (see Fig. 2c,d), along with a good grouping of the three analytical replicates of the NEs. These visual results corroborate the reproducibility of the analyses. and demonstrate that there is no apparent batch effect in either the chromatographic or MS dimensions.

**Fig. 3:**
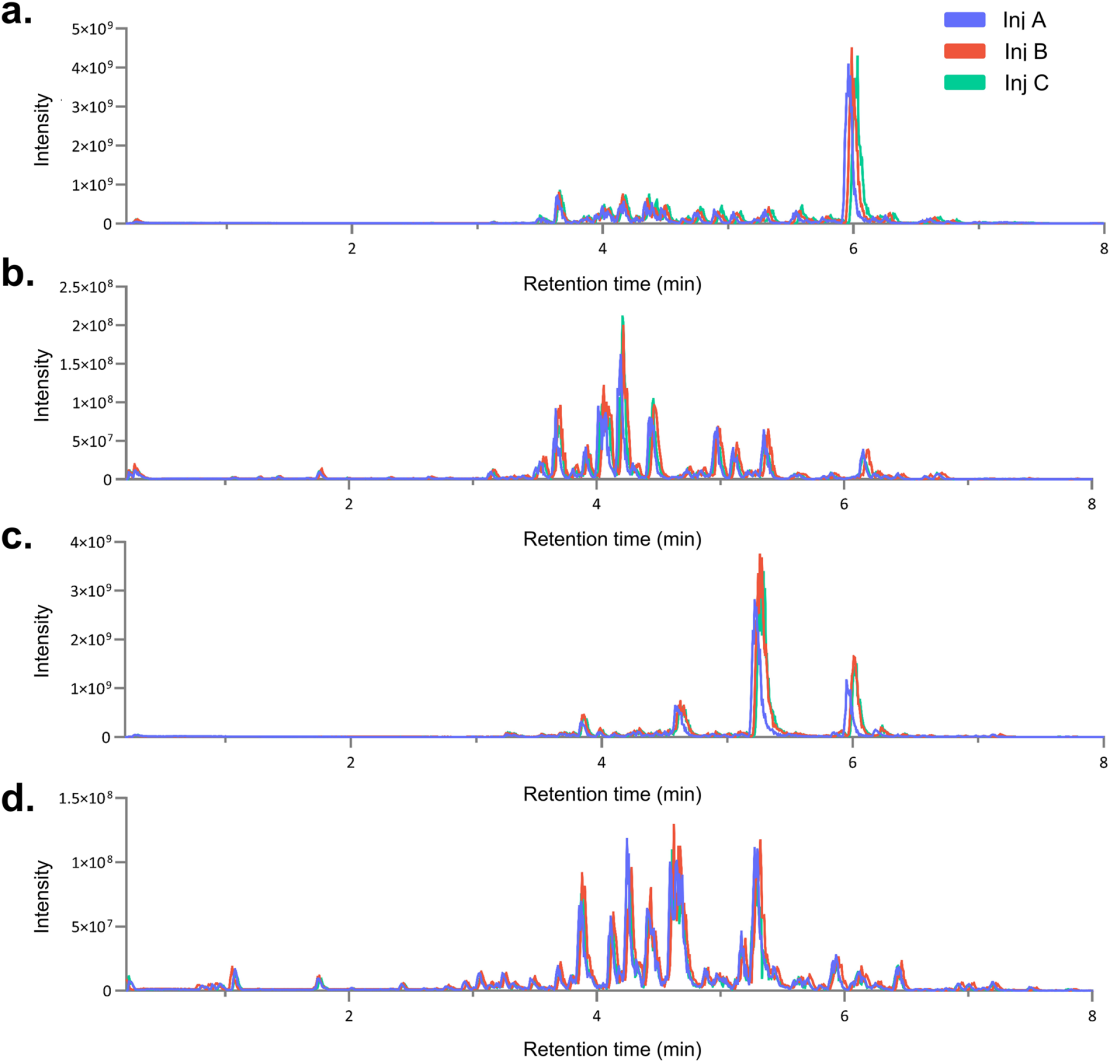
Comparative analysis of UHPLC-HRMS^2^ metabolite profiles across three analytical replicates for the ethyl acetate extracts of *Pristimera indica* roots (PI: panel **a**, NI: panel **b**) and *Tripterygium wilfordii* roots (PI: panel **c**, NI: panel **d**). The color scheme corresponds to the analytical replicates (injections A-C).

Further comparative analyses were conducted on specific samples within the dataset. Figure 3 shows the comparison of UHPLC-HRMS^2^ metabolite profiles across the analytical replicates (injections A-C) for ethyl acetate extracts of *Pristimera. indica* roots (Fig. 3a,b) and *T*ripterygium *wilfordii* roots (Fig. 3c,d). Like the behavior observed in Fig. 2a,b for the QCs, these NEs, in each ionization mode, exhibited slight shifts in retention times, with an ∆RT of 0.05 minutes between injections. The three replicates of both samples were processed and aligned using MZmine3, using identical parameters for the entire dataset (see *Methods*). The outcomes regarding the total detected features in each replicate for both ionization modes are provided in Table 1. The observed number of features between replicates displays a notable similarity. This comprehensive analysis underscores the robustness of the methodology.

**Table 1:** The number of features obtained for the ethyl acetate extract of *Tripterygium wilfordii* roots and *Pristimera indica* roots in the three analytical replicates (inj A-C) in both, PI, and NI modes.

	*T. wilfordii roots*		*P. indica roots*	
	PI	NI	PI	NI
inj A	941	578	992	355
inj B	1021	610	1034	360
inj C	1032	637	1060	386

The data processing was the same for each replicate.

Therefore, to streamline the dereplication procedures, it was concluded that processing and subjecting only the initial analytical replicate (A) to the dereplication pipelines would be adequate. Subsequently, all the forthcoming discussions regarding dereplication results exclusively pertain to the first analytical replicate.

### Overview of the molecular networking and annotation results of the dataset

The processing of the UHPLC-HRMS^2^ metabolite profiling data, construction of the II-FBMN, and subsequent obtention of different putative annotations enabled the assessment of the occurrence of metabolites in the samples, as well as the generation of an overview of the chemical space within the data set. The application of IIMN regrouped the 16,139 nodes, and 13,672 nodes into 14,000 and 10,500 neutral molecules for PI and NI, respectively. While the different annotation workflows are done based on the feature number, the Sirius workflow considered the IIMN results to increase the annotation consistency^38^. Overall, PI mode consistently yielded higher annotation rates compared to NI. Based on the GNPS library search results, the annotation coverage was approximately 11% for PI and 1.5% for NI. In relation to the *in silico* metabolite annotation workflows, the ISDB pipeline achieved annotations of 49% for PI and 40% for NI. Meanwhile, when employing Sirius, the MF prediction coverage stood at 8% for PI and 5% for NI. For structural annotations, Sirius covered 62% in PI and 13% in NI. The numerical results are summarized in Table 2.

**Table 2:** Annotation results overview for the Celastraceae Set in PI and NI modes.

	Positive ionization mode	Negative ionization mode
II-FBMN nodes	16,139	13,103
II-FBMN clusters	1,859	313
II-FBMN Annotation network numbers	3,611	463
GNPS structural annotations	1,751	198
ISDB structural annotations	7,910	5,287
Sirius MF annotations	1,333	726
Sirius structural annotations	9,587	1,545
Canopus chemical classes	9,990	1,744

**Fig. 4:**
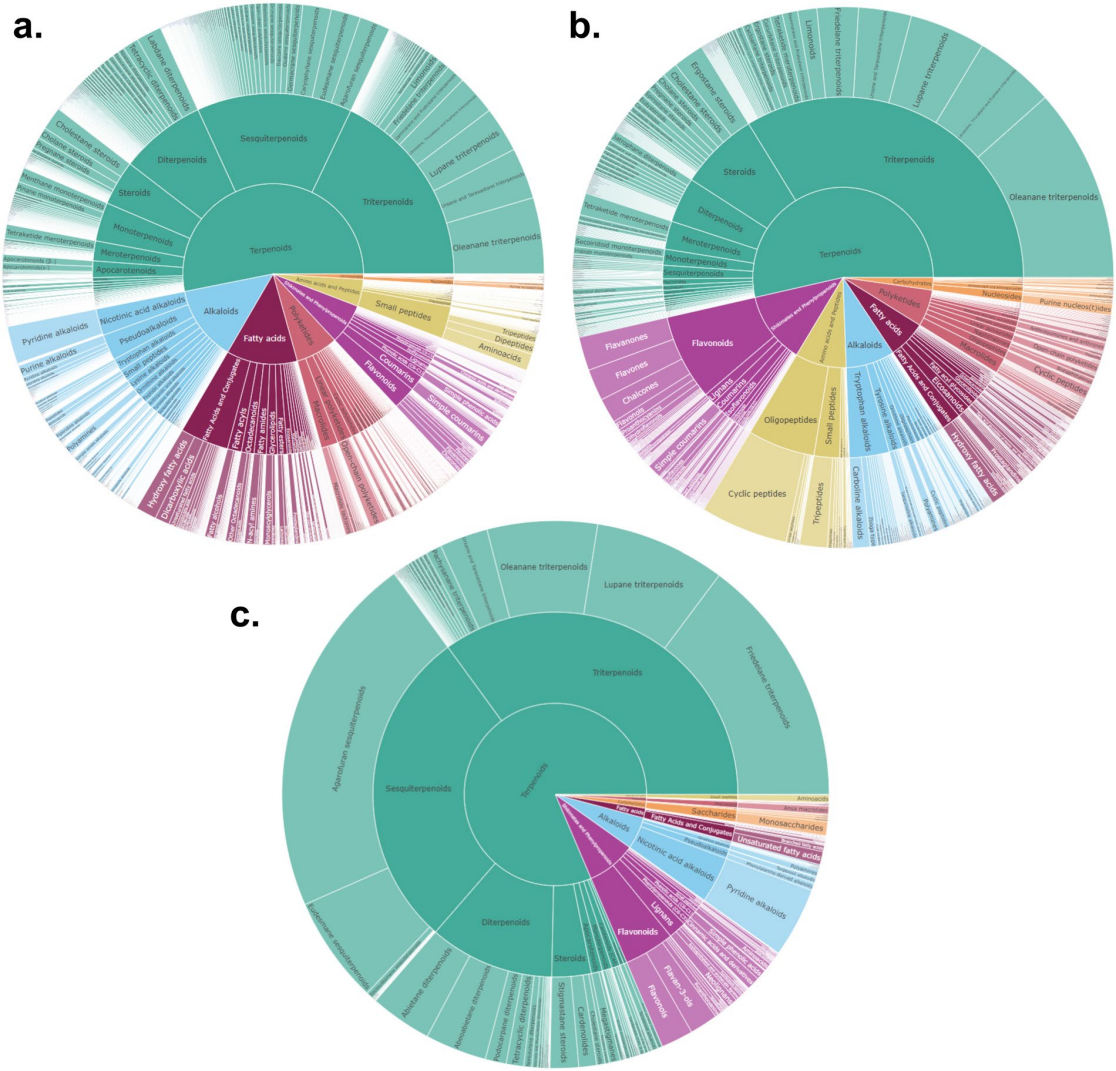
Sunburst representation of the chemical classes proposed by Sirius-Canopus for the (**a**) PI, and (**b**) NI modes. (**c**) Sunburst representation of the chemical classes reported for the Celastraceae family according to Lotus and DNP. Proportions are based on the recurrences of individual classes.

The overviews of the chemical taxonomy derived from Sirius-CANOPUS are shown in the interactive sunburst graphics for PI, and NI modes (see Fig. 4a,b). The proportions of these graphics are based on the recurrences of individual classes in the set. Interestingly, in both ionization modes, the most represented superclasses are derived from the terpenoid pathway, including triterpenoids, sesquiterpenoids, and diterpenoids.

To gain a comprehensive overview and understanding of the chemical class annotation results for the Celastraceae set, a visual comparison was made with the collective set of molecules present in LOTUS and DNP for the Celastraceae family. For this, a sunburst plot of the reported chemical space was generated in the same way done for the CANOPUS chemical classes (Fig. 4c, interactive plot). This showed that the main chemical superclasses included: diterpenes, sesquiterpenes, triterpenes, triterpenoids quinone methides, and maytansinoids. Within each of these superclasses, the main classes correspond to dihydro-*β*-agarofurans, macrolide sesquiterpene alkaloids (macrolactones formed from a dihydro-*β*-agarofuran and a pyridinic dicarboxylic acid), abietanes, friedo-oleanes, celastroloids, and macrolides, respectively. The most represented scaffold corresponds to terpene-like structures, which agrees with the chemical classes proposed by CANOPUS directly from the MS^2^ spectra. This is, as well, consistent with the diverse phytochemical studies that have depicted the general profile of specialized metabolites within the Celastraceae family^4,69–71^.

**Fig. 5:**
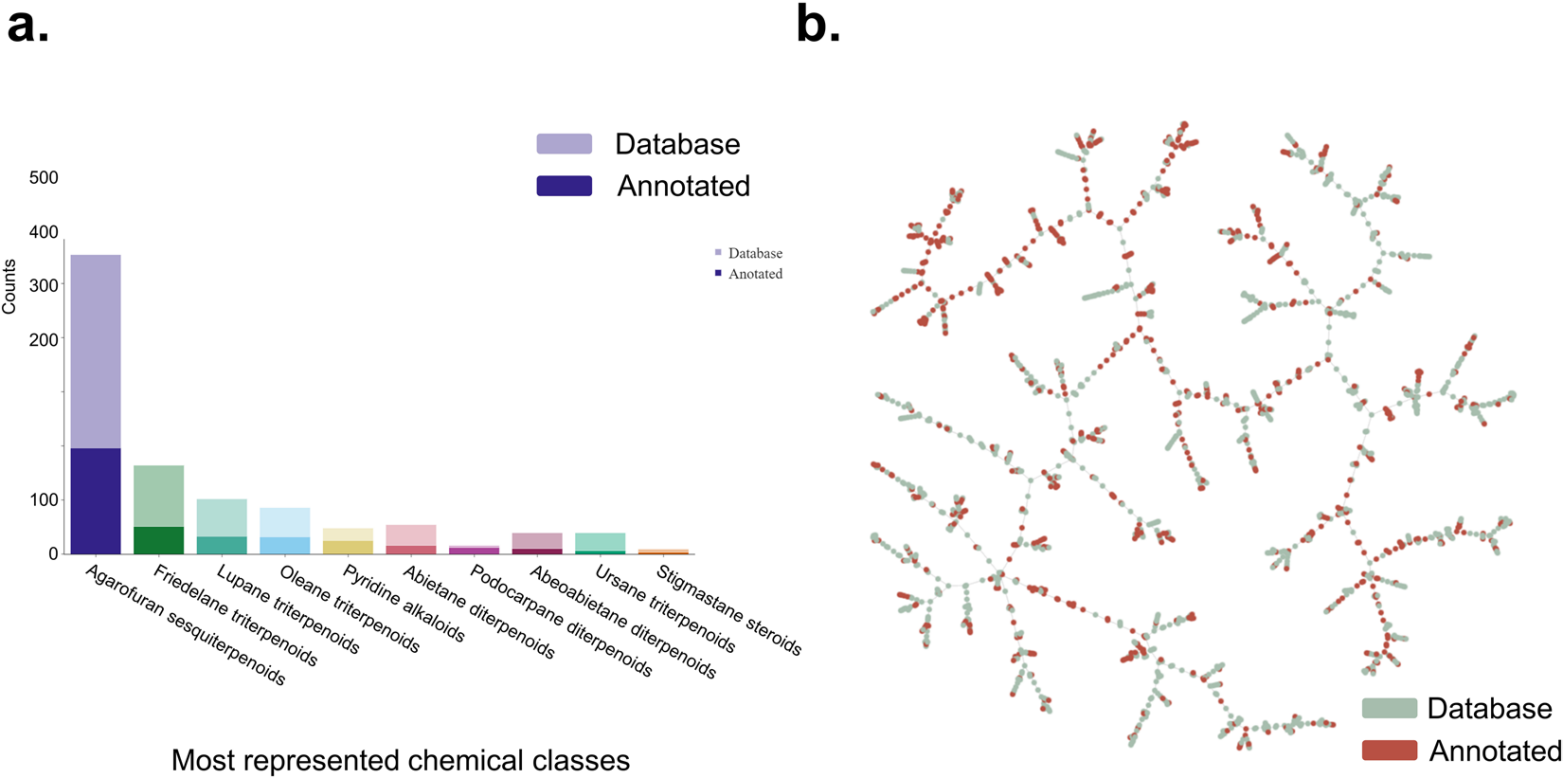
(**a**) Coverage barplot of the 10 most represented chemical classes in the Celastraceae family according to the records present in LOTUS and DNP. Lighter colors represent the total count of molecules in each class in the collective DB, and darker colors represent the count of putatively annotated molecules in each class in the dataset in PI mode. (**b**) TMAP visualization of the overlap between reported chemical space for the Celastraceae family (green) according to the records present in LOTUS and DNP, and the putative annotations for the data set (red) in PI mode.

The distribution of the most represented chemical classes in this botanical family was visualized in Fig. 5a (interactive plot). Since the PI mode data exhibited superior annotation yields, these annotations were employed to determine the coverage proportion for these chemical classes, utilizing structural annotations from the dataset (as shown in Fig. 5a). The most reported compounds are agarofurans sesquiterpenoids, followed by friedelane and lupane triterpenoids. Besides the excellent coverage in terms of chemical classes, the coverage within the most representative individual classes is high.

Additionally, to visualize the annotations and the reported chemical space at the individual molecular structure level, both, were structured using a TMAP visualization^72^. The TMAP forms a minimum spanning tree built to link similar chemical structures using the MAP4 (MinHashed Atom-Pair fingerprint up to 4 bonds) fingerprint^73^. In the diagram of Fig. 5b (interactive plot), each dot represents a chemical structure and is connected to its neighboring dot based on its structural proximity. The color code illustrates the distribution of chemical structures, with green representing the compounds reported in the Celastraceae family (occurring at least once), and red indicating structures annotated in the Celastraceae set. The putative structural annotations are well distributed along the tree, indicating that the set presented here covers a very large part of the chemical space reported for this family so far.

### Evaluation of the annotation results for the Tripterygium wilfordii species

*Tripterygium wilfordii* commonly known as ‘*Thunder God Vine*’ is a fascinating and potent medicinal plant with a long history of traditional use in various Asian cultures, particularly in Chinese herbal medicine^3,13^. Some of the active principles of this plant have captured the interest of modern medicine due to their ability to modulate immune responses and inhibit inflammatory pathways. Consequently, it has become a focal point of research for autoimmune diseases and inflammatory disorders^74,75^. Hence, this species has been extensively studied, as evidenced by the 746 unique entries in this WikiData query (this additional query recovers the individual reports of each compound with the references). To further validate the general workflow used for the Celastraceae set, the annotations obtained based on the MN in PI mode for the extract of *T. wilfordii* roots and bark were assessed in detail.

**Fig. 6:**
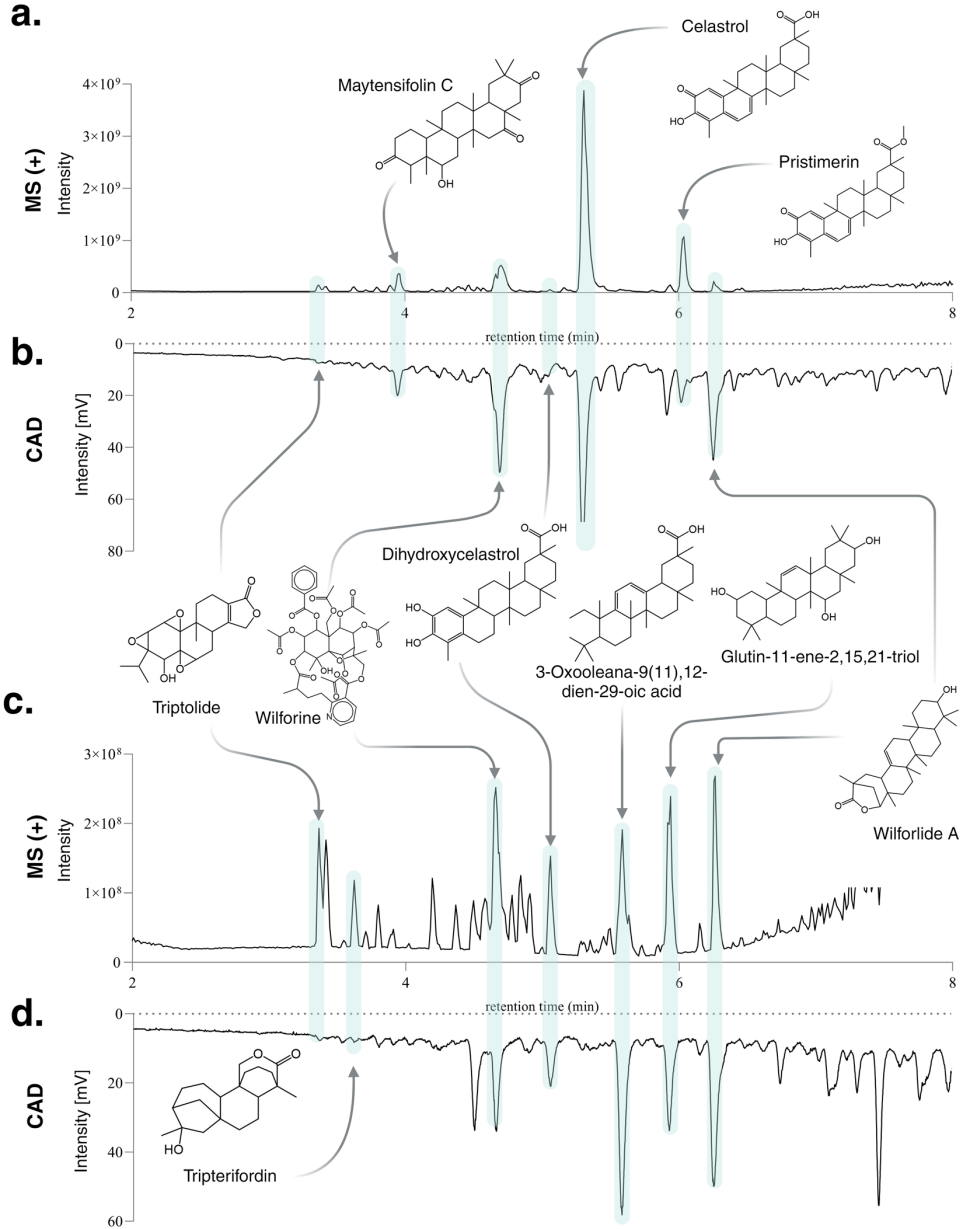
Visualization of the annotation results for the ethyl acetate extract of *Tripterygium wilfordii* **Roots**: (**a**) UHPLC-HRMS PI chromatographic trace. (**b**) Charged Aerosol Detector (CAD) chromatographic trace; and **Stems**: (**c**) UHPLC-HRMS PI chromatographic trace. (**d**) CAD chromatographic trace. Structures are presented in 2D projection.

Based on the overall annotation outcomes, a total of 291 features were successfully annotated for both *T. wilfordii* roots and bark extracts. To visually depict key annotations concerning the primary constituents of *T. wilfordii*, these annotations are summarized in Table 3 and have been presented in Fig. 6. Within this figure, both the MS data and the CAD trace are shown. The comparison of these two traces facilitated a manual assessment of the features corresponding to the major NPs detected in both extracts. The inclusion of the CAD trace not only enhances the dereplication analysis but also aids in the identification of the major constituents^76^. This approach demonstrates a good fitting between the annotation results for these key constituents and existing literature data, reinforcing the notion that these compounds stand as major NPs from the plant. Most of these compounds also corresponded to the most pharmacologically significant ones within the *T. wilfordii* species and *Tripterygium* genus. This includes compounds like celastrol^23,75^, triptolide ^75,77^, and wilforlide A^78^. Additionally, the targeted isolation of celastrol and pristimerin was performed on the root of *T. wilfordii* (data not shown). The structures were confirmed by 1D and 2D NMR analysis. The respective UHPLC-HRMS^2^ analysis confirmed the retention time and the MS^2^ spectral information was shared on the GNPS platform (celastrol, pristimerin). These MSI level 1 annotations and findings contribute to strengthening the confidence in the overall annotation results presented within this study.

**Table 3:** Collective spectral annotations of the ethyl acetate extracts of *Tripterygium wilfordii* roots and bark against the experimental and theoretical spectral DBs in PI mode.

retention time (min)	Row ID	Inchi Key	SpectrumID	Compound Name
3.36	7919	DFBIRQPKNDILPW-UHFFFAOYSA-N	CCMSLIB00000078954	triptolide^75,77^
3.64	5232	KLMZPLYXGZZBCX-UHFFFAOYSA-N	ISDB	tripterifordin^74^
3.94	12233	AQSQZYRCGPMIGT-UHFFFAOYSA-N	ISDB	maytensifolin C^82^
4.57	14904	WQXGLECMNMWOGT-UHFFFAOYSA-N	ISDB	wilforine^83^
5.03	11398	NBMIXMLIGPLJPK-UHFFFAOYSA-N	ISDB	dihydrocelastrol^84^
5.30	10630	KQJSQWZMSAGSHN-UHFFFAOYSA-N	CCMSLIB00005724999	celastrol^23,76^
5.58	10213	HDWVISPHUGFDMW-UHFFFAOYSA-N	ISDB	3-oxooleana-9(11),12-dien-29-oic acid^85^
5.92	10843	LTWRASMKIRWZQN-UHFFFAOYSA-N	ISDB	glutin-11-ene-2,15,21-triol^86^
6.03	11155	JFACETXYABVHFD-UHFFFAOYSA-N	CCMSLIB00004679171	pristimerin^87^
6.27	10215	HHQJBWYXBWOFJY-UHFFFAOYSA-N	ISDB	wilforlide A^78^

InChI Keys correspond to planar (2D) structures.

The comprehensive UHPLC-PDA-CAD-HRMS^2^ metabolite profiling conducted on the set of Celastraceae plants, followed by data processing, facilitated the creation of an extensive set of annotations using state-of-the-art bioinformatic tools. The results of the annotation pipelines were then used to construct a chemical space, which was subsequently compared against the reported chemical space for the Celastraceae family. This work opens avenues for data exploration, enabling the identification of taxonomic relationships based on chemical similarities. Moreover, within the dataset, a subset of features remains unannotated, representing potential connections to novel and uncharacterized NPs. This is exemplified by the work undertaken in the development of *Inventa*^63^. This dataset not only serves as a cornerstone for further investigations into chemotaxonomy but also provides a valuable resource for any research aimed at uncovering the diverse chemical space of this botanical family, known for its abundant array of specialized metabolites.

## Usage Notes

The data set in PI mode was used in the proof of concept to demonstrate the utility of *Inventa*^63^. This is a metabolomic bioinformatic workflow designed to streamline NEs selection with a high potential for containing structurally novel NPs. It bases the computation on the *in-depth* untargeted UHPLC-HRMS^2^ metabolite profiling and annotations results from tools like Sirius. The implementation of *Inventa* on the Celastraceae set highlighted the ethyl acetate extract of the roots of *Pristimera indica* (Willd.) A.C.Sm. (Q11075650) for containing potentially new NPs. In this study, an *in-depth* phytochemical investigation of the *P. indica* roots extracts led to the isolation and characterization of thirteen new *β*-agarofuran compounds (Q104375349), including five of them with a new 9-oxodihydro-*β*-agarofuran base scaffold. Thanks to the inclusion of the MS^2^ spectra of all those compounds in the GNPS experimental DB, and the continuous identification workflow, these identifications are already found in the annotation results for the present set.

The current dataset can be used to describe the specialized chemistry of the set at the species and genus levels. It can be employed to develop chemotaxonomic models since it contains the quality controls and analytical replicates necessary (See Technical validation). Researchers can use the Mass Spec Query Language (MassQL) to interrogate the data set for specific spectral patterns produced by a certain group of compounds^79,80^. For example, this MassQL query searches for the MS^2^ fragments of the 9-oxodihydro-*β*-agarofuran^63^ mentioned above. The results of this query showed that for example the features *m/z* 755.3001 (silviatine A, Q114866936, D ashboard visualization) and *m/z* 713.2905 (silviatine B, Q114866937, Dashboard visualization) were effectively found in the *P. indica* roots extract, from which these compounds were originally described.

Another example could involve searching for the presence of a specific compound of pharmaceutical interest to identify potential biological sources. As an example, the ions for celastrol (Q5057534) were queried in MassQL. The results showed the presence of several precursor ions with a particular MS^2^ fragment at *m/z* 201.09, characteristic of the quinone methide triterpenoids^81^. Several extracts were highlighted for their celastrol content (check the Dashboard comparison). This information enables the contemplation of these sources as potential candidates for isolating the target metabolite and its analogs. Incorporating CAD traces in the provided raw data facilitates a more informed choice when selecting an extract, as it provides a more accurate representation of the NE’s real composition.

## Data Availability

The standard workflow used for processing and generating the Feature-Based Molecular Networking can be found in the GNPS documentation. The scripts used to resolve the taxonomy of the species in the collection are available in this repository: https://github.com/luigiquiros/metadata_preparation. The workflow for ISDB annotation and taxonomical re-weighting is available here: https://taxonomicallyinformedannotation.github.io/tima-r/index.html. The script for cleaning and consolidating the annotations is available here: https://github.com/luigiquiros/inventa. The scripts for the generation of the interactive figures for this Data Descriptor are available in this repository: https://github.com/luigiquiros/Celastraceae-Set-publication-examples. The scripts used to generate the interactive TMAP are available in this repository: https://github.com/mandelbrot-project/pf_1600_datanote/releases/tag/v0.1.
